# Use of Aripiprazole Long Acting Injection in Negative Symptoms of Schizophrenia

**DOI:** 10.1155/2016/7912083

**Published:** 2016-02-15

**Authors:** Suneeta James, Chaya Kapugama, Mohammed Al-Uzri

**Affiliations:** Leicestershire Partnership NHS Trust, Bradgate Mental Health Unit, Groby Road, Leicester, Leicestershire LE3 9EJ, UK

## Abstract

*Background*. Evidence for the efficacious use of second-generation antipsychotics for the treatment of negative symptoms in schizophrenia is scant.* Case Presentation*. We report the case of a 34-year-old female of Afro-Caribbean origin, who presented with prominent negative symptoms of schizophrenia and was successfully treated with aripiprazole long acting injection. Within a period of six to nine months, the patient returned to her premorbid level of functioning.* Conclusion*. Aripiprazole long acting injection promises benefits in the treatment of negative symptoms of schizophrenia. Further research needs to be conducted on the use of this drug.

## 1. Background 

Negative symptoms of schizophrenia contribute to poor functional outcomes [1] and poorer quality of life [2] for patients. Increased disability associated with negative symptoms may also lead to increased burden on caregivers [3]. Negative symptoms tend to persist longer and have proven to be more difficult to treat than positive symptoms. Few second-generation antipsychotics are known to be more efficacious in treatment of negative symptoms than first-generation antipsychotics [4] but their effect size is medium to small [5]. There are many small randomized controlled trials (RCTs) showing varying levels of efficacy for different second-generation oral antipsychotic drugs [6–9].

Aripiprazole prolonged-release suspension for injection is a new preparation licensed for maintenance treatment of schizophrenia in adults whose condition has been stabilized with oral aripiprazole. It was launched in the UK in January 2014.

We present the case of a thirty-four-year-old female with prominent negative symptoms of schizophrenia successfully treated with aripiprazole long acting injection.

## 2. Case Presentation 

A 34-year-old Afro-Caribbean female was admitted to a psychiatric inpatient unit with worsening social withdrawal, apathy, and poor independent functioning for the past two years. She was known to psychiatric services in 2012. At that time the patient had a brief admission when she displayed aggression and persecutory delusions believing that the police were having sex with her and that she was being recorded through cameras. She was not treated with psychotropic medications at the time. From the records, her symptoms appear to have remitted spontaneously and she was discharged. She was lost to follow-up thereafter.

The patient is from Zimbabwe and was granted asylum status approximately ten years previously after coming to the UK. She was employed with good social functioning. Following her first admission, she lost her job and her accommodation due to not paying rent.

The patient was living at a hostel for the homeless when she was seen by her current team in 2014 and had become increasingly reliant on staff for her basic needs. Her self-care was poor and apathy was felt to be the prominent feature. She had very limited funds available to her and was not claiming state benefits. Her interaction was very limited. Staff, at her hostel, had not witnessed any behavior indicative of positive psychotic symptoms and there were no incidences of aggression or violence. She was referred to the Assertive Outreach community team but she did not engage with the team.

On this second admission the patient was noted to have poor self-care, weight loss, and poor motivation. There were no objective symptoms of clinical depression and the patient denied ongoing low mood. She did not engage with staff and refused physical examination, blood tests, and oral medication. Positive and Negative Syndrome Scale (PANSS) [10] scoring was completed, yielding 18, 36, and 64 on the positive scale, the negative scale, and the general psychopathology scale, respectively. The patient was commenced on aripiprazole long acting injection. Prior to this she had had two doses of aripiprazole short acting intramuscular injection without any adverse effects.

Around one month after her first dose the patient started showing improvement by interacting with staff, going for walks and taking an interest in her state benefits. She agreed to do blood tests including an infection screen and they were within normal limits. Magnetic resonance imaging of the brain showed mild focal atrophy of parietal and occipital lobes. A detailed cognitive examination was completed using the Repeatable Battery for the Assessment of Neuropsychological Status (RBANS) [11] and Behavioral Assessment of Dysexecutive Syndrome [12] and no deficits were found.

Within the third month of this episode, the patient was transferred to a specialist psychiatric rehabilitation unit. Over the next six months she showed steady improvement and reached her premorbid functional level. She had begun cooking and shopping for herself. She started attending a computer course. Other aspects of her management included occupational therapy and psychology inputs. Repeated PANSS evaluation was done, with 7, 13, and 25 on the positive scale, the negative scale, and the general psychopathology scale, respectively.

## 3. Conclusion 

Aripiprazole is a second-generation antipsychotic medication that is licensed for use in schizophrenia spectrum disorders. It is unique in its mechanism of action due to its partial agonism at dopamine D2 receptors compared to other second-generation antipsychotic drugs. The efficacy of oral aripiprazole in treatment of schizophrenia is well established [13, 14]. Aripiprazole is known to improve both positive and negative symptoms of schizophrenia [15].

US Food and Drug Administration approved aripiprazole long acting injection in 2013. The manufacturer's advice was 400 mg once monthly dose administered intramuscularly, for initiation and maintenance treatment, and concurrent treatment with oral aripiprazole during the first fourteen days [16]. A 52-week, multicenter, randomized, double-blind, placebo-controlled study using this long acting preparation concluded that it appears to be a well-tolerated maintenance treatment for schizophrenia [17]. However the evidence around long acting aripiprazole injection is still scarce.

Our patient presented with predominant negative symptoms following a decline from her premorbid level of functioning. We decided to prescribe aripiprazole as it had less risk of developing extrapyramidal and metabolic side effects compared to other atypical antipsychotics. Since the patient refused oral aripiprazole, we commenced her on the long acting injection without the two-week period of oral drug as recommended.

The patient showed significant improvement in all areas of functioning after commencement of long acting aripiprazole injection. Our patient presented in the early stages of her illness and the total duration of her illness, at the time, amounted to no more than two years. In addition, she also underwent rigorous rehabilitation during the treatment period. Aripiprazole was the first antipsychotic to be prescribed for her.

The use of aripiprazole long acting injection in prominent negative symptoms seems promising. Further studies need to be conducted to evaluate the benefits of this long acting injection in negative symptoms of schizophrenia.
