# Corrigendum to “Use of Aripiprazole Long Acting Injection in Negative Symptoms of Schizophrenia”

**DOI:** 10.1155/2019/9595638

**Published:** 2019-09-16

**Authors:** Suneeta James, Chaya Kapugama, Mohammed Al-Uzri

**Affiliations:** Leicestershire Partnership NHS Trust, Bradgate Mental Health Unit, Groby Road, Leicester, Leicestershire LE3 9EJ, UK

In the article titled “Use of Aripiprazole Long Acting Injection in Negative Symptoms of Schizophrenia” [1], there was an error in the Abstract as the patient was mistakenly described to be of “Afro-Caribbean origin” and once again as an “Afro-Caribbean female” in the “2. Case Presentation” section. The authors apologize for that mistake as the patient should be referred to as “a female of African descent”.
